# Type 2 Diabetes Coagulopathy Proteins May Conflict With Biomarkers Reflective of COVID-19 Severity

**DOI:** 10.3389/fendo.2021.658304

**Published:** 2021-06-25

**Authors:** Abu Saleh Md Moin, Ahmed Al-Qaissi, Thozhukat Sathyapalan, Stephen L. Atkin, Alexandra E. Butler

**Affiliations:** ^1^ Diabetes Research Center (DRC), Qatar Biomedical Research Institute (QBRI), Hamad Bin Khalifa University (HBKU), Qatar Foundation (QF), Doha, Qatar; ^2^ Academic Endocrinology, Diabetes and Metabolism, Hull York Medical School, Hull, United Kingdom; ^3^ Department of Endocrinology, Leeds Medical School, Leeds, United Kingdom; ^4^ Research Department, Royal College of Surgeons in Ireland Bahrain, Adliya, Bahrain

**Keywords:** type 2 diabetes, hypoglycemia, COVID-19, biomarkers, proteomics

## Abstract

**Objective:**

Detailed proteomic analysis in a cohort of patients with differing severity of COVID-19 disease identified biomarkers within the complement and coagulation cascades as biomarkers for disease severity has been reported; however, it is unclear if these proteins differ sufficiently from other conditions to be considered as biomarkers.

**Methods:**

A prospective, parallel study in T2D (n = 23) and controls (n = 23). A hyperinsulinemic clamp was performed and normoglycemia induced in T2D [4.5 ± 0.07 mmol/L (81 ± 1.2 mg/dl)] for 1-h, following which blood glucose was decreased to ≤2.0 mmol/L (36 mg/dl). Proteomic analysis for the complement and coagulation cascades were measured using Slow Off-rate Modified Aptamer (SOMA)-scan.

**Results:**

Thirty-four proteins were measured. At baseline, 4 of 18 were found to differ in T2D *versus* controls for platelet degranulation [Neutrophil-activating peptide-2 (p = 0.014), Thrombospondin-1 (p = 0.012), Platelet factor-4 (p = 0.007), and Kininogen-1 (p = 0.05)], whilst 3 of 16 proteins differed for complement and coagulation cascades [Coagulation factor IX (p < 0.05), Kininogen-1 (p = 0.05), and Heparin cofactor-2 (p = 0.007)]; STRING analysis demonstrated the close relationship of these proteins to one another. Induced euglycemia in T2D showed no protein changes *versus* baseline. At hypoglycemia, however, four proteins changed in controls from baseline [Thrombospondin-1 (p < 0.014), platelet factor-4 (p < 0.01), Platelet basic protein (p < 0.008), and Vitamin K-dependent protein-C (p < 0.00003)], and one protein changed in T2D [Vitamin K-dependent protein-C, (p < 0.0002)].

**Conclusion:**

Seven of 34 proteins suggested to be biomarkers of COVID-19 severity within the platelet degranulation and complement and coagulation cascades differed in T2D *versus* controls, with further changes occurring at hypoglycemia, suggesting that validation of these biomarkers is critical. It is unclear if these protein changes in T2D may predict worse COVID-19 disease for these patients.

**Clinical Trial Registration:**

https://clinicaltrials.gov/, identifier NCT03102801.

## Introduction

Proteomic pathways of platelet degranulation and the complement and coagulation cascades have recently been suggested as biomarkers of disease severity, being derived from a cohort of patients with differing severity of COVID-19 disease, including non-survivors (1). These data were in accord with another recently reported plasma proteomic analysis that also identified biological pathways involved in platelet degranulation and the coagulation cascade (2) in COVID-19 disease. Biomarkers to predict COVID-19 disease progression and outcome that allow a measured and appropriate proactive intervention may be key in improving patient treatment (3); however, they do need to be distinct from levels found in an uninfected population. In this regard, protein expression showing altered platelet function resulting in a prothrombotic potential together with changed markers of coagulation (4) has been reported in patients with type 2 diabetes (T2D) (5). With the increasing prevalence of diabetes, particularly with those patients having a worse COVID-19 outcome, this is of particular relevance (6).

Therefore, we undertook platelet degranulation and complement and coagulation cascade proteomic analysis in subjects with and without T2D to compare with these coagulopathy pathways described recently using proteomics in COVID-19 disease (1).

## Methods

Type 2 diabetes (T2D) (n = 23) and control subjects (n = 23) were enrolled in a case-controlled study, approved by Yorkshire and Humber Research Ethics Committee. A hyperinsulinemic clamp was performed as reported (7); all subjects were Caucasian and underwent a 10-h fast prior to the clamp. T2D: T2D baseline glucose 7.6 ± 0.4 mmol/L (136.8 ± 7.2 mg/dl), reduced to 4.5 ± 0.07 mmol/L (81 ± 1.2 mg/dl) for 1-h, following which the blood glucose was decreased to ≤2.0 mmol/L (36 mg/dl). Controls: baseline 4.9 ± 0.1 mmol/L (88.2 ± 1.8 mg/dl) (**Supplementary Figure 1**). The duration of diabetes was <10 years and all T2D subjects were on a stable dose of medication (metformin, statin, and/or angiotensin converting enzyme inhibitor/angiotensin receptor blocker) over the prior 3 months. For those with T2D, no medications for glycemic control except metformin was allowed. All had normal renal and hepatic biochemical indices and no prior history of cancer nor any contraindication to insulin infusion to achieve hypoglycemia (ischemic heart disease, epilepsy, seizure history, drop attacks, history of adrenal insufficiency, and treated hypothyroidism).

Proteins that were described for platelet degranulation (18 of 27 proteins) and the complement and coagulation cascades (16 of 19 proteins) (1) were measured using the Slow Off-rate Modified Aptamer (SOMA)-scan plasma protein measurement (7), shown in **Table 1**.

**Table 1:** Demographic and clinical characteristics of the study participants.

Baseline	Type 2 Diabetes (n = 23)	Controls (n = 23)	p-value
Age (years)	64 ± 8	60 ± 10	<0.0001
Sex (M/F)	12/11	11/12	0.77
Weight (kg)	90.9 ± 11.1	79.5 ± 8.8	<0.0001
Height (cm)	167 ± 14	169 ± 5	0.64
BMI (kg/m^2^)	32 ± 4	28 ± 3	<0.0001
Systolic BP (mmHg)	132 ± 8	122 ± 8	0.001
Diastolic BP (mmHg)	81 ± 7	75 ± 6	0.003
Duration of diabetes (years)	4.5 ± 2.2	N/A	
HbA1c (mmol/mol)	51.2 ± 11.4	37.2 ± 2.2	<0.0001
HbA1c (%)	6.8 ± 1.0	5.6 ± 0.2	<0.0001

BMI, Body mass index; BP, Blood pressure; HbA1c, Hemoglobin A1c; N/A, not applicable.

### Statistical Analysis

There are no studies detailing the changes in platelet degranulation or complement and coagulation proteins in response to hypoglycemia on which to base a power calculation. Sample size for pilot studies has been reviewed by Birkett and Day (8). They concluded that a minimum of 20 degrees-of-freedom was required to estimate effect size and variability. Hence, we needed to analyze the samples from a minimum of 20 patients per group. Data trends were visually evaluated for each parameter and non-parametric tests were applied on data that violated the assumptions of normality when tested using the Kolmogorov-Smirnov Test. Comparison between groups was performed at each timepoint using Student’s t-test. A p-value of <0.05 was considered statistically significant. Within-group comparisons are as follows: changes from baseline, and from hypoglycemia, to each subsequent timepoint were compared using Student’s t-test. The sample size was too small to adjust for baseline covariates. Statistical analysis was performed using Graphpad Prism (San Diego, CA, USA).

For the proteomic analysis we fitted an intercept-free general linear model as a function of a subgroup (i.e. condition:timepoint), while taking the patient ID as a random effect using the R package limma. Subsequently, we computed the p value for two contrasts: baseline to hypoglycemia for both T2D and controls, and false discovery rate (FDR) corrected at a value of <0.05 as the cutoff for significance.

## Results

As reported previously (5), T2D subjects had higher BMI (p = 0.001) and higher blood pressure (p < 0.003) with a duration of diabetes 4.5 ± 2.2 years (**Table 1**).

For the 46 protein biomarkers described by Shu et al. (1), 34 were available for measurement in the Somalogic platform: 4 of 18 were found to differ at baseline in T2D for platelet degranulation [Neutrophil-activating peptide 2 (NAP-2) (p = 0.014), Thrombospondin-1 (THBS1) (p = 0.012), Platelet factor 4 (PF4) (p = 0.007), and Kininogen-1 (KNG1) (p = 0.05)], whilst 3 of 16 proteins differed for the complement and coagulation cascades [Coagulation factor IX (F9) (p < 0.05), Kininogen-1 (KNG1) (p = 0.05), and Heparin cofactor 2 (SERPIND1) (p = 0.007)] (**Table 2**). All proteins that differed between T2D and controls were higher at baseline in T2D, apart from Kininogen-1 that was lower at baseline in T2D.

**Table 2:** Proteins identified as being altered in COVID-19 disease categorized according to **(A)** platelet degranulation; **(B)** complement and coagulation cascades in non-COVID infected type 2 diabetes and control subjects at baseline.

A. PLATELET DEGRANULATION				
Target Full Name	Target	UniProt	Entrez Gene Symbol	T-test Baseline Control *vs* T2D
Transgelin-2	Transgelin-2	P37802	TAGLN2	0.608
Neutrophil-activating peptide 2	NAP-2	P02775	PPBP	0.014
Fibrinogen gamma chain	Fibrinogen g-chain dimer	P02679	FGG	0.364
Fibrinogen	Fibrinogen	P02671, P02675 P02679	FGA	0.333
Thrombospondin-1	Thrombospondin-1	P07996	THBS1	0.012
Platelet factor 4	PF-4	P02776	PF4	0.007
von Willebrand factor	vWF	P04275	VWF	0.986
Serpin peptidase inhibitor, clade A (alpha-1 antiproteinase, antitrypsin), member 3	alpha-1-antichymotrypsin complex	P01011	SERPINA3	0.892
Fructose-bisphosphate aldolase A	aldolase A	P04075	ALDOA	0.377
Metalloproteinase inhibitor 1	TIMP-1	P01033	TIMP1	0.079
Plasminogen	Plasminogen	P00747	PLG	0.398
Kininogen-1	“Kininogen, HMW”	P01042	KNG1	0.05
Alpha-2-antiplasmin	a2-Antiplasmin	P08697	SERPINF2	0.277
Fibronectin	Fibronectin	P02751	FN1	0.995
CD109 antigen	CD109	Q6YHK3	CD109	0.074
Alpha-2-macroglobulin	a2-Macroglobulin	P01023	A2M	0.924
Kallistatin	Kallistatin	P29622	SERPINA4	0.079
Apolipoprotein A-I	Apo A-I	P02647	APOA1	0.466
B. COMPLEMENT AND COAGULATION CASCADES				
Target Full Name	Target	UniProt	Entrez Gene Symbol	T-test Baseline Control *vs* T2D
Fibrinogen gamma chain	Fibrinogen g-chain dimer	P02679	FGG	0.364
Fibrinogen	Fibrinogen	P02671, P02675 P02679	FGA	0.333
Complement factor I	Factor I	P05156	CFI	0.132
von Willebrand factor	vWF	P04275	VWF	0.986
Coagulation factor IX	Coagulation Factor IX	P00740	F9	0.049
Complement factor H	Factor H	P08603	CFH	0.675
Complement component C9	C9	P02748	C9	0.993
Vitronectin	Vitronectin	P04004	VTN	0.294
Plasminogen	Plasminogen	P00747	PLG	0.398
Kininogen-1	“Kininogen, HMW”	P01042	KNG1	0.05
Alpha-2-antiplasmin	a2-Antiplasmin	P08697	SERPINF2	0.277
Complement component C4B		P0C0L4 P0C0L5	C4B	0.337
Plasma serine protease inhibitor	PCI	P05154	SERPINA5	0.608
Heparin cofactor 2	Heparin cofactor II	P05546	SERPIND1	0.007
Vitamin K-dependent protein C	Protein C	P04070	PROC	0.50
Alpha-2-macroglobulin	a2-Macroglobulin	P01023	A2M	0.924

All proteins that differed between T2D and controls were higher at baseline in T2D apart from Kininogen-1 that was lower in T2D.

Students’ t-test was used to determine differences between protein levels. Proteins that differed significantly (p < 0.05) are shown in red font. Proteins that are common to both the platelet degranulation and the complement/coagulation cascades are shaded in orange.

**Figure 1:**
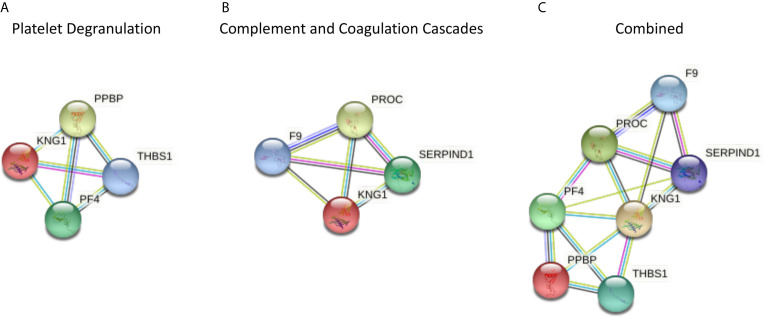
The protein-protein interaction tool STRING 11.0 (Search Tool for the Retrieval of Interacting Genes) was used to visualize the significantly different proteins in type 2 diabetes (T2D) compared to controls, and for all of the proteomic proteins in COVID-19 disease severity described by others (1) (https://string-db.org/). Interactions between proteins are evidence-based and collated from databases, experiments, neighborhood, gene fusion, co-occurrence, text mining, co-expression, and homology. Here, we determined the relationships between the platelet degranulation **(A)** and complement and coagulation cascade proteins **(B)** presented in the study by Shu et al. (1) that were significantly different between non-COVID infected T2D and control subjects. **(A)** Platelet degranulation proteins that differed significantly between T2D and control subjects, indicating their relationship to one another. **(B)** Complement and coagulation cascade proteins that differed significantly between T2D and control subjects, indicating their relationship to one another. **(C)** Combined platelet degranulation and complement and coagulation cascade proteins that differed significantly in T2D, indicating their relationships to one another.

Those proteins that significantly differed between T2D and controls share a close relationship to one another, as shown by the protein-protein interaction tool STRING (Search Tool for the Retrieval of Interacting Genes) pathways (**Figure 1**).

Following the hyperinsulinemic clamp inducing euglycemia in T2D, no difference from baseline to euglycemia was seen for any of the proteins (data not shown). However, when the clamp was continued to hypoglycemia, four proteins changed in controls from baseline [Thrombospondin-1 (p < 0.014), platelet factor 4 (p < 0.01), Platelet basic protein (p < 0.008), and Vitamin K-dependent protein C (p < 0.00003)], but only one in T2D [Vitamin K-dependent protein C (p < 0.0002)].

In view of the discrepancy in BMI between T2D and control subjects, correlations with the individual proteins *versus* BMI were undertaken. Only Coagulation factor IX was positively correlated with BMI, in T2D subjects only (p = 0.04, r = 0.43) (**Supplementary Figure 2**).

## Discussion

From the potential biomarkers identified by Shu for platelet degranulation and the complement and coagulation cascades that were purported to be predictors of severity in COVID-19 disease (1), 7 of the 34 proteins that were available in the SOMA-scan proteomic platform differed at baseline in patients with T2D compared to controls. This would potentially suggest that T2D patients with COVID-19 disease would be classed as having more severe disease, as some of their protein biomarker levels would be already altered, potentially leading to the introduction of early intensive therapy that may not be warranted or appropriate. Therefore, the significant difference seen in T2D compared to controls indicates the need for validation of such markers in the non-COVID-19 infected T2D population before they can be considered as biomarkers for COVID-19 and its severity. Conversely, that these biomarkers already differ in T2D may predict that more severe COVID-19 disease will result and be an indicator that more proactive intervention is required, as patients with T2D have a known propensity for increased COVID-19 disease severity (5).

In a wider context, exclusive of COVID-19, the results of this study also show that the complement and coagulation proteins investigated were not affected by glycemic changes until frank hypoglycemia was induced, showing that glycemic variability that is known to affect proteins through oxidative stress was not an issue here (9).

Neutrophil-activating peptide 2 is a heparin sulfate degrading enzyme (10) basally increased in T2D and its elevation is seen in acute coronary syndrome and it is thought to have a role in platelet-mediated vascular inflammation (11).

Thrombospondin-1 is a component of extracellular matrix that acts as a negative regulator of angiogenesis and is associated with cardiovascular disease where its elevation is seen in cardiac insults such as myocardial infarction (12). Here, it was basally increased in T2D *versus* controls that is in accord with its elevation and association with induced delay in re-endothelialization following arterial injury in diabetes (13), likely through increased inflammation (14) that is seen in T2D.

Platelet factor 4 was increased basally in T2D, as has been reported previously, and has been shown to be discriminatory for diabetes nephropathy and neuropathy (15).

Kininogen-1 plays a role in the kallikrein-kinin system (cooperating with the renin-angiotensin system); it has been suggested as a possible urinary biomarker for diabetic nephropathy (16) and may effect renal protection (17). Here, basal Kininogen-1 was higher in controls than T2D and this has been reported by others where it was suggested that its reduction may reflect bradykinin formation that has potent inflammatory effects (18). Taken together, this data suggests that basal elevation in T2D for Neutrophil-activating peptide 2, Thrombospondin-1, and Platelet factor 4 and a reduction in Kininogen-1 may contribute towards the vascular pathology of T2D.

Coagulation factor IX is a result of activation of the intrinsic coagulation pathway leading to fibrin formation and is not reported to be different in T2D *versus* controls, the converse to what was observed here (19); however, its elevation in T2D may potentially increase the risk of a vascular event. Notably, this was the only factor that correlated positively with BMI, indicating that obesity may be a factor in its expression.

Heparin cofactor 2 is a vascular protective factor that primarily acts as an anticoagulant (20) and, in this context, its elevation would be protective.

Hypoglycemia resulted in alteration of the complement and coagulation cascades with an increase in thrombospondin, platelet factor 4, platelet basic protein (NAP-2 or PPBP), and vitamin K-dependent protein C. Vitamin K-dependent protein C functions as a natural anticoagulant to downregulate thrombin generation in the clotting cascade with cardioprotective properties (21), and therefore its elevation shown here may be seen to be protective.

These data show that protein changes in response to glucose variability above and below the physiological range and back to normoglycemia do occur but, to date, have scarcely been investigated despite their potential importance.

It is unclear from the Shu report what differing therapeutic agents were used for each of the COVID-19 severity categories (1), and therefore whether these treatment regimes may affect protein expression. This may be particularly relevant for hydroxychloroquine therapy, that has been associated with hypoglycemia in both diabetes and non-diabetes patients (22), and here we show that proteomic biomarker changes may result from hypoglycemia particularly in those without diabetes where four proteins changed in controls whilst only one protein differed from baseline in T2D.

Limitations of the study include that the SOMAlogic panel only included 34 of the 46 proteins in the platelet degranulation and complement and coagulation cascades that were reported by Shu et al. (1), and that the proteomic analysis used in each study differed so direct comparison to the Shu study (1) or others (2) was not possible. Of note, however, most proteins were common to the differing proteomic platforms used. In addition, as all study subjects were Caucasian, the results presented here may not be generalizable to other ethnic groups.

In conclusion, 8 of 34 protein biomarkers contained within the platelet degranulation and complement and coagulation cascades differed in T2D, with further changes at hypoglycemia that may be modified by COVID-19 therapy, suggesting that validation of these biomarkers is critical. It is unclear if these protein changes in T2D may be predictors of more severe COVID-19 disease in these patients.

## Data Availability Statement

The raw data supporting the conclusions of this article will be made available by the authors, without undue reservation.

## Ethics Statement

The studies involving human participants were reviewed and approved by the Newcastle & North Tyneside Ethics committee. The patients/participants provided their written informed consent to participate in this study.

## Author Contributions

AM and AB analyzed the data and wrote the manuscript. AA-Q contributed to study design, performed experiments, collected, analyzed, and interpreted data, and edited the manuscript. TS supervised clinical studies and edited the manuscript. SA contributed to study design, data interpretation, and the writing of the manuscript. All authors contributed to the article and approved the submitted version. AB is the guarantor of this work.

## Conflict of Interest

The authors declare that the research was conducted in the absence of any commercial or financial relationships that could be construed as a potential conflict of interest.

## Supplementary Material

Supplementary Figure 1 Comparison of blood glucose levels in plasma before, during, and after iatrogenic induction of hypoglycemia. Blood sampling was performed at baseline (BL), at hypoglycemia (0 min), and post-hypoglycemia (30 min, 1-h, 2-h, 4-h, and 24-h) for controls (white circles) and for T2D (black squares). At baseline (BL), blood glucose was 7.5 ± 0.4 mmol/L for T2D subjects and 5.0 ± 0.1 mmol/L for control subjects. At point of hypoglycemia, blood glucose was 2.0 ± 0.03 mmol/L for the T2D cohort and 1.8 ± 0.05 mmol/L for the control cohort. Statistics: ****p < 0.0001, control *vs* T2D.

Supplementary Figure 2 Correlation of Coagulation factor IX with BMI. Coagulation factor IX showed a positive correlation with BMI in subjects with type 2 diabetes (T2D) (p = 0.04, r = 0.43). No such correlation was seen in the control cohort.

## References

[1] Shu T Ning W Wu D Xu J Han Q Huang M. Plasma Proteomics Identify Biomarkers and Pathogenesis of COVID-19. Immunity (2020) 53(5):1108–22.e5. 10.1016/j.immuni.2020.10.008 33128875 PMC7574896

[2] Overmyer KA Shishkova E Miller IJ Balnis J Bernstein MN Peters-Clarke TM. Large-Scale Multi-Omic Analysis of COVID-19 Severity. Cell Syst (2021) 12(1):23–40.e7. 10.1016/j.cels.2020.10.003 33096026 PMC7543711

[3] Kahal H Aburima A Spurgeon B Wraith KS Rigby AS Sathyapalan T. Platelet Function Following Induced Hypoglycaemia in Type 2 Diabetes. Diabetes Metab (2018) 44:431–6. 10.1016/j.diabet.2018.04.004 29784564

[4] Sobczak AIS Stewart AJ. Coagulatory Defects in Type-1 and Type-2 Diabetes. Int J Mol Sci (2019) 20(24):6345. 10.3390/ijms20246345 PMC6940903 31888259

[5] Moin ASM Al-Qaissi A Sathyapalan T Atkin SL Butler AE. Do Biomarkers of COVID-19 Severity Simply Reflect a Stress Response in Type 2 Diabetes: Biomarker Response to Hypoglycemia. Metabolism (2021) 114:154417. 10.1016/j.metabol.2020.154417 33157081 PMC7609242

[6] Agrawal D Jaiswal P Goyanka B. Diabetes and Covid-19: A Review. Int J Res Pharm Sci (2020) 11(1). 10.26452/ijrps.v11iSPL1.2729

[7] Kahal H Halama A Aburima A Bhagwat AM Butler AE Grauman J. Effect of Induced Hypoglycemia on Inflammation and Oxidative Stress in Type 2 Diabetes and Control Subjects. Sci Rep (2020) 10(1):4750. 10.1038/s41598-020-61531-z 32179763 PMC7075968

[8] Birkett MA Day SJ. Internal Pilot Studies for Estimating Sample Size. Stat Med (1994) 13(23-24):2455–63. 10.1002/sim.4780132309 7701146

[9] Ceriello A Monnier L Owens D. Glycaemic Variability in Diabetes: Clinical and Therapeutic Implications. Lancet Diabetes Endocrinol (2019) 7(3):221–30. 10.1016/S2213-8587(18)30136-0 30115599

[10] Hoogewerf AJ Leone JW Reardon IM Howe WJ Asa D Heinrikson RL. CXC Chemokines Connective Tissue Activating Peptide-III and Neutrophil Activating Peptide-2 Are Heparin/Heparan Sulfate-Degrading Enzymes. J Biol Chem (1995) 270(7):3268–77. 10.1074/jbc.270.7.3268 7852412

[11] Smith C Damås JK Otterdal K Øie E Sandberg WJ Yndestad A. Increased Levels of Neutrophil-Activating Peptide-2 in Acute Coronary Syndromes: Possible Role of Platelet-Mediated Vascular Inflammation. J Am Coll Cardiol (2006) 48(8):1591–9. 10.1016/j.jacc.2006.06.060 17045893

[12] Zhang K Li M Yin L Fu G Liu Z. Role of Thrombospondin−1 and Thrombospondin−2 in Cardiovascular Diseases. Int J Mol Med (2020) 45(5):1275–93. 10.3892/ijmm.2020.4507 PMC7138268 32323748

[13] Ii M Takenaka H Asai J Ibusuki K Mizukami Y Maruyama K. Endothelial Progenitor Thrombospondin-1 Mediates Diabetes-Induced Delay in Reendothelialization Following Arterial Injury. Circ Res (2006) 98(5):697–704. 10.1161/01.RES.0000209948.50943.ea 16484619

[14] Lopez-Dee Z Pidcock K Gutierrez LS. Thrombospondin-1: Multiple Paths to Inflammation. Mediators Inflamm (2011) 2011:296069. 10.1155/2011/296069 21765615 PMC3134184

[15] Fritschi J Christe M Lämmle B Marbet G Berger W Duckert F. Platelet Aggregation, β-Thromboglobulin and Platelet Factor 4 in Diabetes Mellitus and in Patients With Vasculopathy. Thromb Haemost (1984) 52(06):236–9. 10.1055/s-0038-1661186 6241750

[16] Nakorn PN Pannengpetch S Isarankura-Na-Ayudhya P Thippakorn C Lawung R Sathirapongsasuti N. Roles of Kininogen-1, Basement Membrane Specific Heparan Sulfate Proteoglycan Core Protein, and Roundabout Homolog 4 as Potential Urinary Protein Biomarkers in Diabetic Nephropathy. EXCLI J (2020) 19:872–91. 10.17179/excli2020-1396 PMC7355151 32665774

[17] Bodin S Chollet C Goncalves-Mendes N Gardes J Pean F Heudes D. Kallikrein Protects Against Microalbuminuria in Experimental Type I Diabetes. Kidney Int (2009) 76(4):395–403. 10.1038/ki.2009.208 19516248

[18] Sharma JN Al-Shoumer KA Matar KM Al-Gharee HY Madathil NV. Bradykinin-Forming Components in Kuwaiti Patients With Type 2 Diabetes. Int J Immunopathol Pharmacol (2013) 26(3):699–705. 10.1177/039463201302600313 24067466

[19] Barillari G Fabbro E Pasca S Bigotto E. Coagulation and Oxidative Stress Plasmatic Levels in a Type 2 Diabetes Population. Blood Coagulation Fibrinol an Int J Haemostasis Thromb (2009) 20(4):290–6. 10.1097/MBC.0b013e328329e49b 19318924

[20] Kurahashi K Inoue S Yoshida S Ikeda Y Morimoto K Uemoto R. The Role of Heparin Cofactor II in the Regulation of Insulin Sensitivity and Maintenance of Glucose Homeostasis in Humans and Mice. J Atheroscl Thromb (2017) 24(12):1215–30. 10.5551/jat.37739 PMC5742367 28502917

[21] Ren D Giri H Li J Rezaie AR. The Cardioprotective Signaling Activity of Activated Protein C in Heart Failure and Ischemic Heart Diseases. Int J Mol Sci (2019) 20(7):1762. 10.3390/ijms20071762 PMC6479968 30974752

[22] Salman Mardones P Quevedo Langenegger I Arias Thormann M Stehr Gesche C Bancalari Selman A. Hypoglycemia Due to Hydroxychloroquine, an Uncommon Association But to Keep in Mind, Case Report and Review of Literature. J Diabetes Metab Disord Control (2020) 7(1):6–7.

