# Supplementary figures and images for: Type 2 Diabetes Coagulopathy Proteins May Conflict With Biomarkers Reflective of COVID-19 Severity
Source: Front Endocrinol (Lausanne). 2021 Jun 25;12:658304. doi: 10.3389/fendo.2021.658304 (PMC8267927; doi:10.3389/fendo.2021.658304)

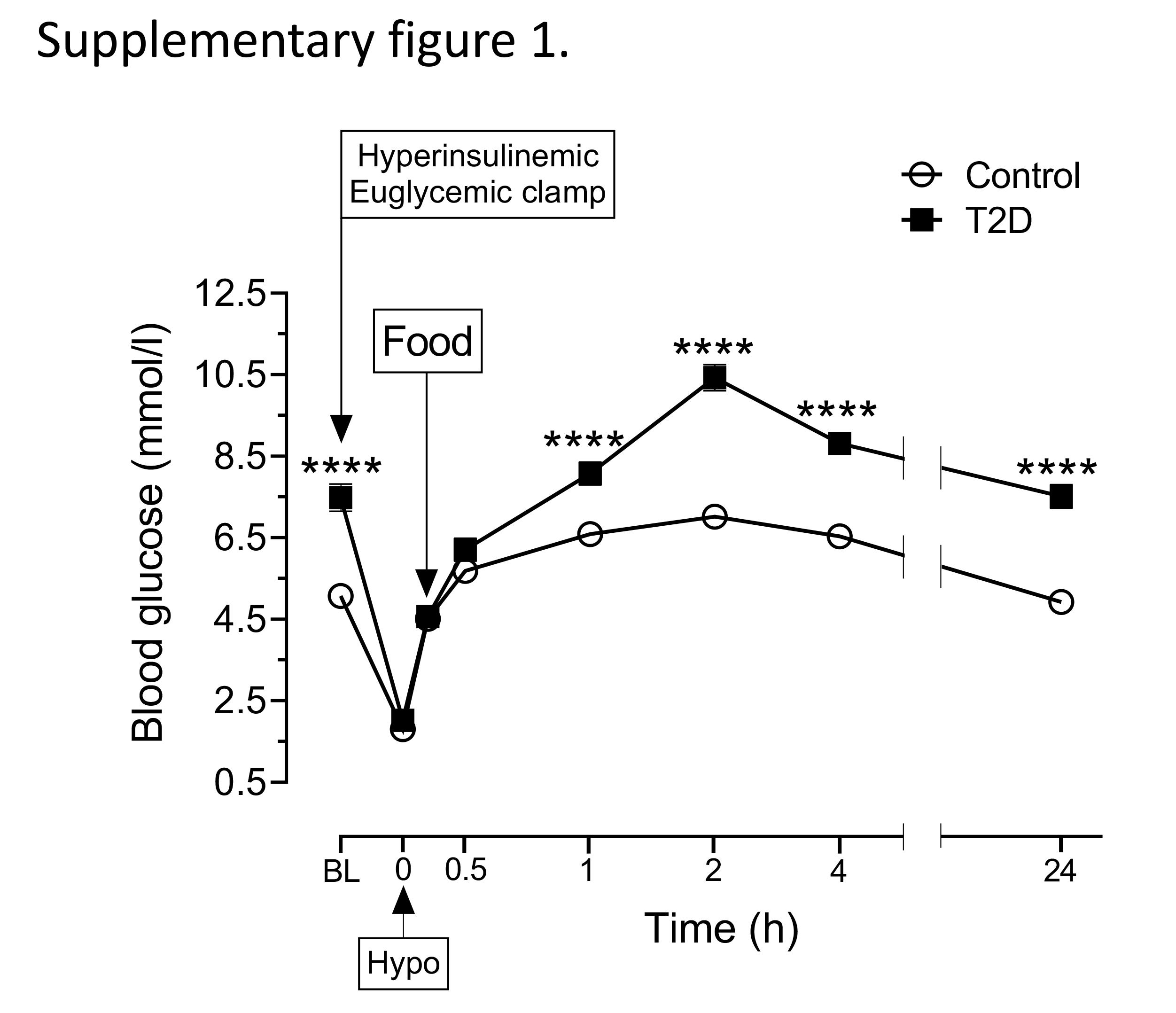

Supplement: Supplementary Figure 1 — Comparison of blood glucose levels in plasma before, during, and after iatrogenic induction of hypoglycemia. Blood sampling was performed at baseline (BL), at hypoglycemia (0 min), and post-hypoglycemia (30 min, 1-h, 2-h, 4-h, and 24-h) for controls (white circles) and for T2D (black squares). At baseline (BL), blood glucose was 7.5 ± 0.4 mmol/L for T2D subjects and 5.0 ± 0.1 mmol/L for control subjects. At point of hypoglycemia, blood glucose was 2.0 ± 0.03 mmol/L for the T2D cohort and 1.8 ± 0.05 mmol/L for the control cohort. Statistics: ****p < 0.0001, control vs T2D. [file Image_1.tif]

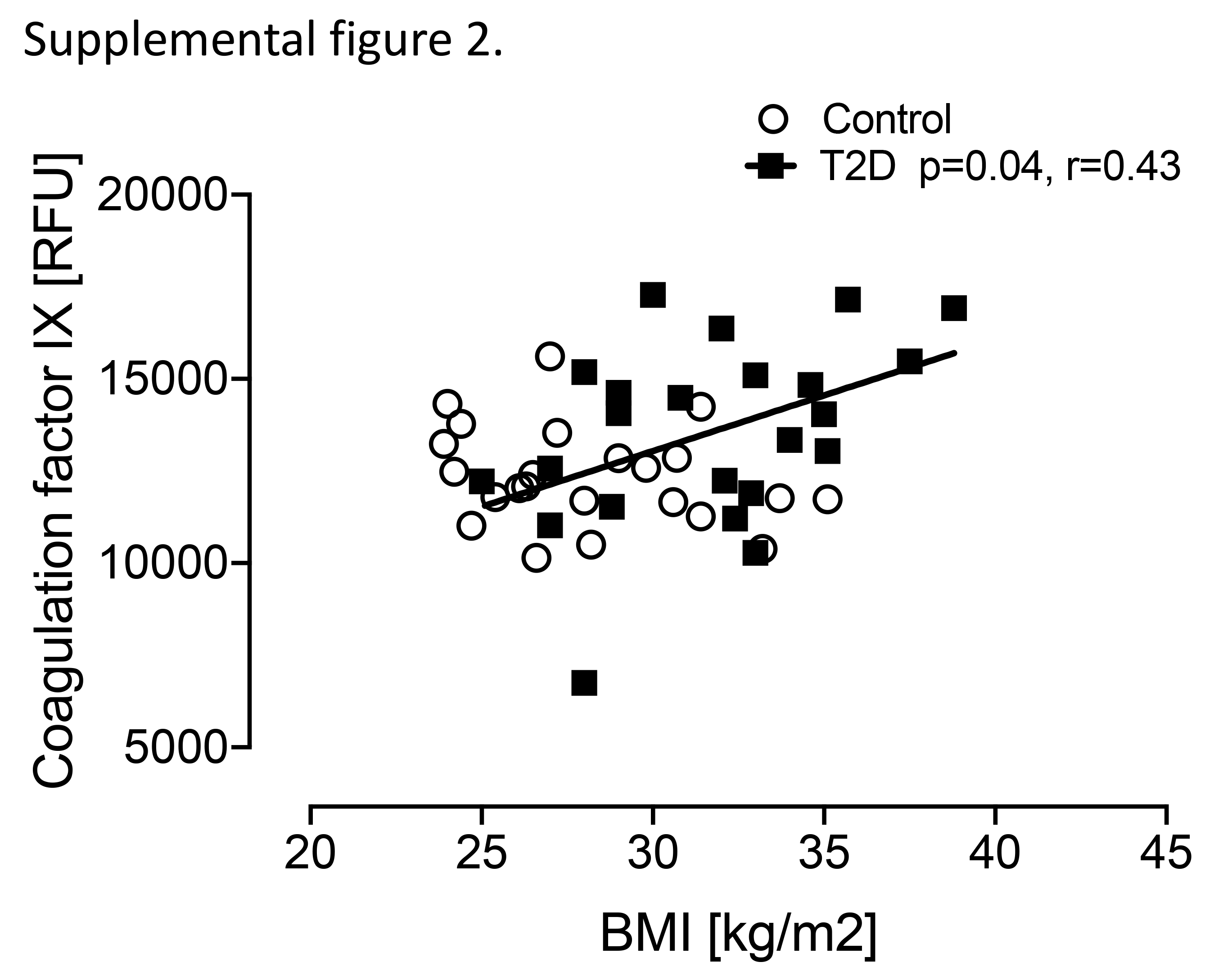

Supplement: Supplementary Figure 2 — Correlation of Coagulation factor IX with BMI. Coagulation factor IX showed a positive correlation with BMI in subjects with type 2 diabetes (T2D) (p = 0.04, r = 0.43). No such correlation was seen in the control cohort. [file Image_2.tif]
